# High-Molecular-Weight Hyaluronic Acid Inhibits IL-1β-Induced Synovial Inflammation and Macrophage Polarization through the GRP78-NF-κB Signaling Pathway

**DOI:** 10.3390/ijms222111917

**Published:** 2021-11-03

**Authors:** Chien-Hsing Lee, Chi-Fu Chiang, Feng-Chih Kuo, Sheng-Chiang Su, Chia-Luen Huang, Jhih-Syuan Liu, Chieh-Hua Lu, Chang-Hsun Hsieh, Chih-Chien Wang, Chian-Her Lee, Pei-Hung Shen

**Affiliations:** 1 National Defense Medical Center, Division of Endocrinology and Metabolism, Tri-Service General Hospital, Taipei 114, Taiwan; doc10383@gmail.com (C.-H.L.); shoummie@hotmail.com (F.-C.K.); doc10504@gmail.com (S.-C.S.); gallon828@hotmail.com (C.-L.H.); ajleonn21@hotmail.com.tw (J.-S.L.); undeca2001@gmail.com (C.-H.L.); 10324@yahoo.com.tw (C.-H.H.); 2 National Defense Medical Center, School of Dentistry, Taipei 114, Taiwan; dofugood1205@outlook.com; 3 National Defense Medical Center, Department of Orthopedics, Tri-Service General Hospital, Taipei 114, Taiwan; tsghcc@gmail.com; 4 Department of Orthopedics, Taipei Medical University, Taipei 110, Taiwan; chianherlee@yahoo.com.tw

**Keywords:** osteoarthritis, hyaluronic acid, GRP78, synoviocytes, macrophage

## Abstract

Recent evidence has suggested that synovial inflammation and macrophage polarization were involved in the pathogenesis of osteoarthritis (OA). Additionally, high-molecular-weight hyaluronic acid (HMW-HA) was often used clinically to treat OA. GRP78, an endoplasmic reticulum (ER) stress chaperone, was suggested to contribute to the hyperplasia of synovial cells in OA. However, it was still unclear whether HMW-HA affected macrophage polarization through GRP78. Therefore, we aimed to identify the effect of HMW-HA in primary synovial cells and macrophage polarization and to investigate the role of GRP78 signaling. We used IL-1β to treat primary synoviocytes to mimic OA, and then treated them with HMW-HA. We also collected conditioned medium (CM) to culture THP-1 macrophages and examine the changes in the phenotype. IL-1β increased the expression of GRP78, NF-κB (p65 phosphorylation), IL-6, and PGE2 in primary synoviocytes, accompanied by an increased macrophage M1/M2 polarization. GRP78 knockdown significantly reversed the expression of IL-1β-induced GRP78-related downstream molecules and macrophage polarization. HMW-HA with GRP78 knockdown had additive effects in an IL-1β culture. Finally, the synovial fluid from OA patients revealed significantly decreased IL-6 and PGE2 levels after the HMW-HA treatment. Our study elucidated a new form of signal transduction for HMW-HA-mediated protection against synovial inflammation and macrophage polarization and highlighted the involvement of the GRP78-NF-κB signaling pathway.

## 1. Introduction

Osteoarthritis (OA) is the most common reason for total hip and knee replacement [1]. The mechanisms leading to the progression of cartilage degradation and failure have been extensively investigated [2]. Recently, some research has proposed that chronic low-grade inflammation is the most common pathogenesis of OA. In addition, synovitis is now recognized as a characteristic of OA in both its early and late stages and a result of activated macrophages infiltrating the synovial membrane [3]. One widely used treatment option is the intra-articular injection of hyaluronic acid (HA) to replace the synovial fluid that has lost its viscoelastic properties [4]. In osteoarthritic joints, the synovial fluid always contains a lower concentration of HA than healthy joints do [5]. In inflammation and tissue injury, HA plays a central role because it can function as a pro- and anti-inflammatory molecule [6]. In addition, studies have reported that high-molecular-weight (HMW)-HA inhibited the production of proinflammatory mediators [7]. HA binding to ICAM-1 down regulates NF-kB, which, in turn, decreases the production of IL-6 [8]. Other studies have shown that HA modulates inflammation based on its different molecular weight [9]. HMW-HA reduced inflammation in experimental arthritis and in interleukin (IL)-1β-stimulated synovial fibroblasts [10].

OA is characterized by an excessive production of several inflammatory mediators [11]. Among these mediators is the proinflammatory cytokine IL-1β, which is synthesized locally by synoviocytes and chondrocytes [12] and has been shown to play a central role in the cartilage damage sustained in arthritis by creating an imbalance between cartilage degradation and repair processes [13]. Several studies have shown that macrophages are the most predominant immune cells in OA [14,15]. Low-grade inflammation may promote the polarization of macrophages into the M1 phenotype in an inflamed synovium [16]. These subpopulations can be generally categorized as classically activated macrophages (M1 macrophages), which are a subset of proinflammatory macrophages induced by proinflammatory mediators such as lipopolysaccharide and interferon-γ [17]. M1 macrophages have an enhanced proinflammatory cytokine production, such as IL-12 and tumor necrosis factor (TNF)-α [18]. Alternatively, activated macrophages (M2 macrophages) are generated by anti-inflammatory mediators such as IL-4 and IL-13. M2 macrophages have high levels of anti-inflammatory cytokines IL-10 and transforming growth factor (TGF)-β [18]. Moreover, HMW-HA suppresses macrophage M1 polarization and enhances the IL-10 production of JNK and NF-κB [19]. The transcription factor NF-κB regulates multiple aspects of innate and adaptive immune functions [20], and plays a critical role in regulating the survival, activation and polarization of the macrophages [21]. NF-κB pathways are involved in the M1 polarization of the RAW 264.7 macrophage and blocking the activation of NF-κB signaling can promote the polarization of M1 macrophages to M2 macrophages [20]. In addition, NF-κB transcription factors regulate inflammatory responses to cytokines such as IL-6 and prostaglandin E2 (PGE2), which are the main catabolic factors involved in OA [22]. However, no study has clearly elucidated the role of HMW-HA in macrophage polarization in synovial inflammation.

Endoplasmic reticulum (ER) stress is a cellular danger signal that is triggered by a failure to fold synthesized ER proteins. The ER stress response has attracted attention for its role in OA cartilage [23]. To help proteins fold properly, ER contains a master molecular chaperone, GRP78 (BIP) [23]. When protein folding is disturbed, GRP78 synthesis is increased, which subsequently binds GRP78 to misfolded proteins to prevent them from forming aggregates and assists them in refolding [24]. Therefore, it is suggested that GRP78 expression contributes to several human diseases, including OA and rheumatoid arthritis (RA). Additionally, an abnormal GRP78 response to ER stress contributes to the inflammation of the synoviocytes and leads to cartilage destruction in RA [25]. Some studies have also indicated that the upregulation of GRP78 is associated with OA [26]. Although the expression and roles of GRP78 have been investigated in OA, the response of synoviocyte inflammation to ER stress has not been clarified.

In the present study, we used IL-1β to treat synoviocytes to mimic OA. We then collected conditional medium (CM) from synoviocytes and used it to culture THP-1 cells to examine the change in the macrophage phenotype. Furthermore, we used a knockdown of GRP78 in synoviocytes and combined HMW-HA treatment to elucidate the role of GRP78 in OA-related synovial inflammation and macrophage polarization.

## 2. Results

### 2.1. IL-1β Treatment Increased Inflammatory Responses and GRP78 Expression

**Figure 1:**
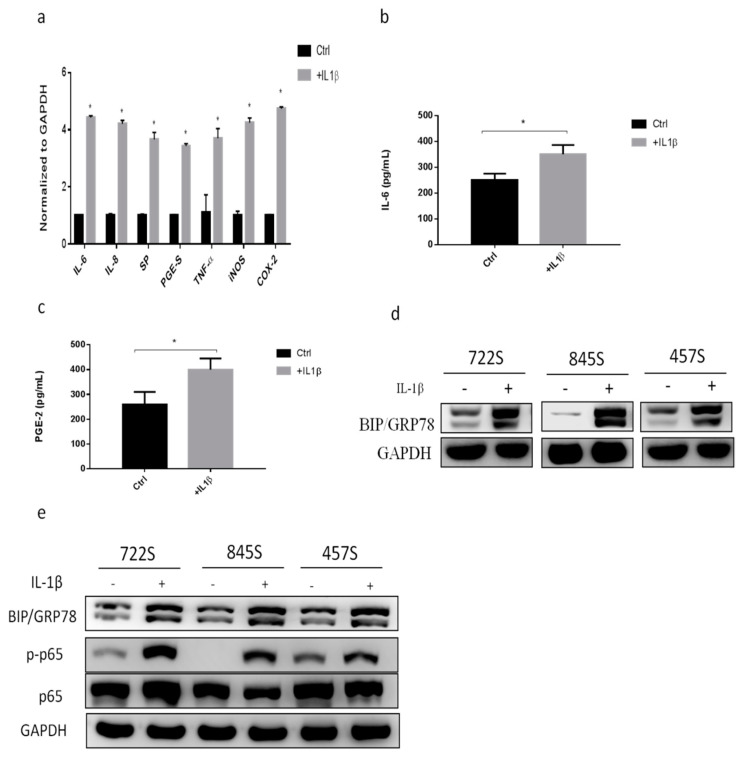
Effects of IL-1β on the inflammatory factors and GRP78 in primary synoviocytes of OA patients. Primary synoviocytes were treated with IL−1β (10 ng/mL) for 24 h. (**a**) Quantitative PCR analysis of IL−6, IL−8, SP, PGE−S, TNF−α, iNOS, and COX2. (**b**,**c**) ELISA analysis of IL−6 and PGE2. (**d**,**e**) Western blot analysis of GRP78, p65 phosphorylation, and p65. Data are expressed as the mean ± SD, * *p* < 0.05. The data presented are representative of three independent experiments.

IL-1β is thought to be one of the most important cytokines in the OA inflammatory response. We used IL-1β (10 ng/mL) to treat synoviocytes to mimic the OA condition. To evaluate the expression of inflammatory factors during synovial inflammation, we used the primary synoviocytes from OA patients and then treated them with IL-1β for 24 h. The levels of inflammatory genes IL-6, IL-8, substance P (SP), PGE synthase (PGE-S), TNF-α, inducible nitric oxide synthase (iNOS), and cyclooxygenase 2 (COX2) were significantly increased in the group treated with IL-1β compared with controls (Figure 1a). In addition, IL-6 and PGE2 are important proinflammatory cytokines in OA. The levels of cytokine IL-6 and PGE2 in synoviocytes were significantly increased in the group treated with IL-1β (Figure 1b,c), suggesting that IL-1β treatment can induce the inflammatory state of the synovium. Next, we evaluated the expression of GRP78 during synovial inflammation. We found that GRP78 expression was increased in the synoviocytes treated with IL-1β (Figure 1d). In the setting of degenerative knee OA, we examined the NF-κB signaling pathway and the inflammatory-associated signaling of p65, which is a subunit of the NF-κB transcription complex that plays a crucial role in biological processes such as inflammation. The phosphorylation of p65 was increased in the group treated with IL-1β (Figure 1e). These findings indicated that IL-1β treatment increased the expressions of inflammatory factors and GRP78.

### 2.2. IL-1β-Treated Synoviocytes Affected the Polarization of Macrophages

**Figure 2:**
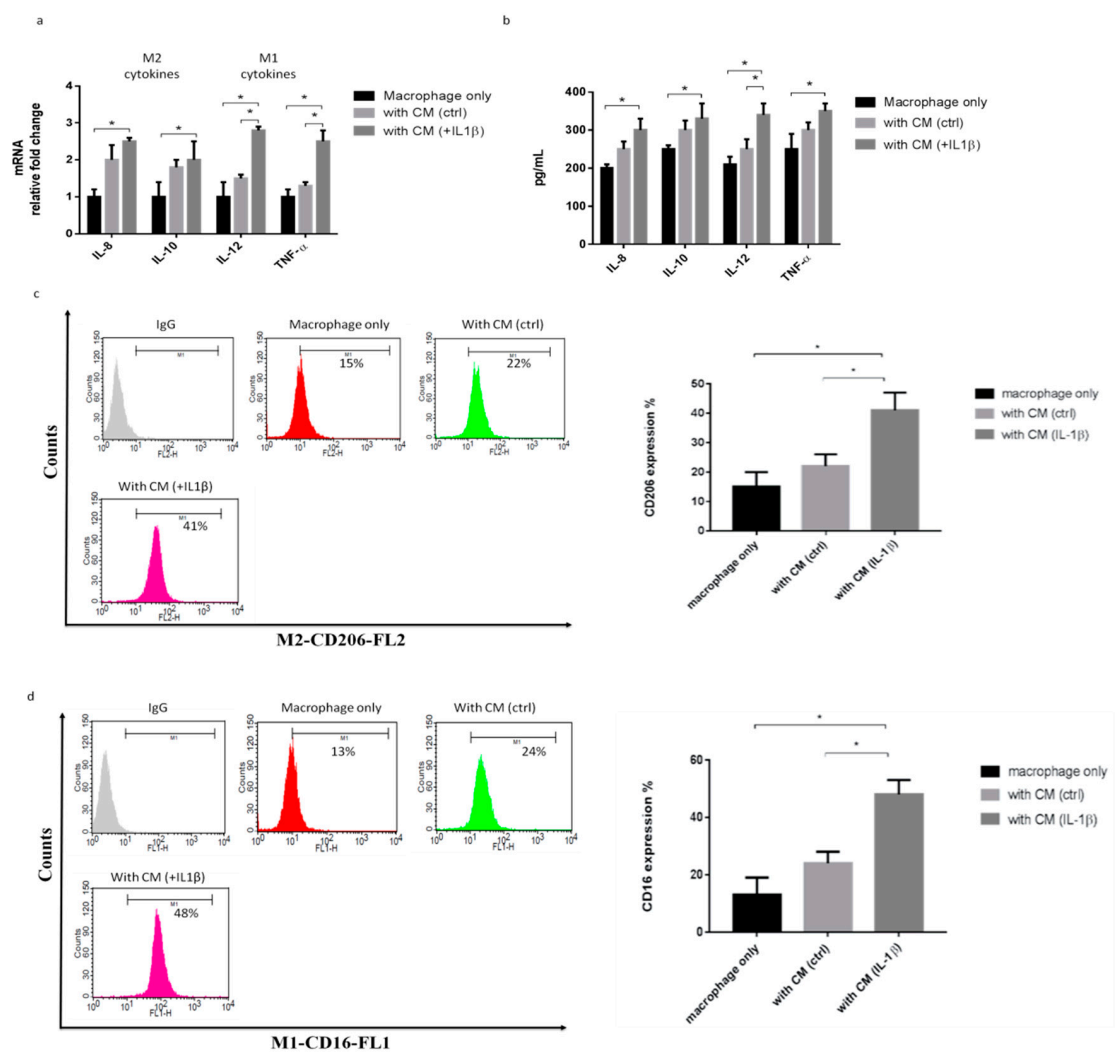
IL-1β-treated primary synoviocytes affected M1/M2 polarization. The primary synoviocytes were treated with IL-1β (10 ng/mL) for 24 h and then replaced with fresh medium; 24 h later, the CM was collected to treat THP-1 cells. (**a**) Quantitative PCR analysis of IL-8, IL-10, IL-12, and TNF-α. (**b**) ELISA analysis of IL-8, IL-10, IL-12, and TNF-α. (**c**,**d**) Flow cytometry analysis of the expression of CD206, a marker of M2, and the expression of CD16, a marker of M1. Data are expressed as the mean ± SD, * *p* < 0.05. IgG as a negative control. The data presented are representative of three independent experiments.

To explore the role of the macrophage phenotype in the OA condition, we collected the supernatant from synoviocytes as conditioned media (CM). CM were then harvested and used to treat macrophages, and the macrophage phenotypes were then examined. Previous research has identified the specific markers expressed by the M2 cytokine markers (IL-8 and IL-10) and the M1 cytokine markers (IL-12 and TNF-α). The M2 and M1 markers were significantly increased in cells polarized with CM plus IL-1β compared with the CM group (Figure 2a). Similarly, the M2 and M1 cytokines were also significantly increased in cells polarized with CM plus IL-1β compared with the CM group (Figure 2b). Flow cytometric analysis was employed to detect the surface markers of the macrophage phenotypes. The expressions of CD206 (an M2 surface marker) and CD16 (an M1 surface marker) were increased by cells polarized with CM plus IL-1β, compared with the CM group (Figure 2c,d). These results showed that the IL-1β treatment increased inflammation in synoviocytes and also increased macrophage M1 and M2 polarization.

### 2.3. Knockdown GRP78 in Synoviocytes Decreased Inflammatory Responses and Promoted M2 Polarization

**Figure 3:**
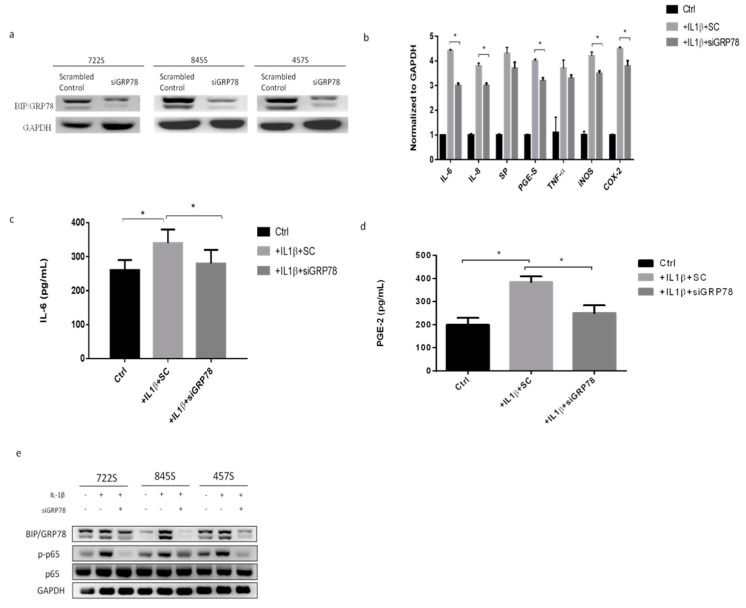
Knockdown of GRP78 decreased inflammatory factors and associated inflammatory signaling, using knockdown GRP78 in IL−1β (10 ng/mL) treated synoviocytes. (**a**) GRP78 knockdown efficiency in synoviocytes. SC, scrambled control. (**b**) Quantitative PCR analysis of IL−6, IL−8, SP, PGE−S, TNF−α, iNOS, and COX2. (**c**,**d**) ELISA analysis of IL−6 and PGE2. (**e**) Western blot analysis of GRP78, p65 phosphorylation, and p65. Data are expressed as the mean ± SD, * *p* < 0.05. The data presented are representative of three independent experiments.

**Figure 4:**
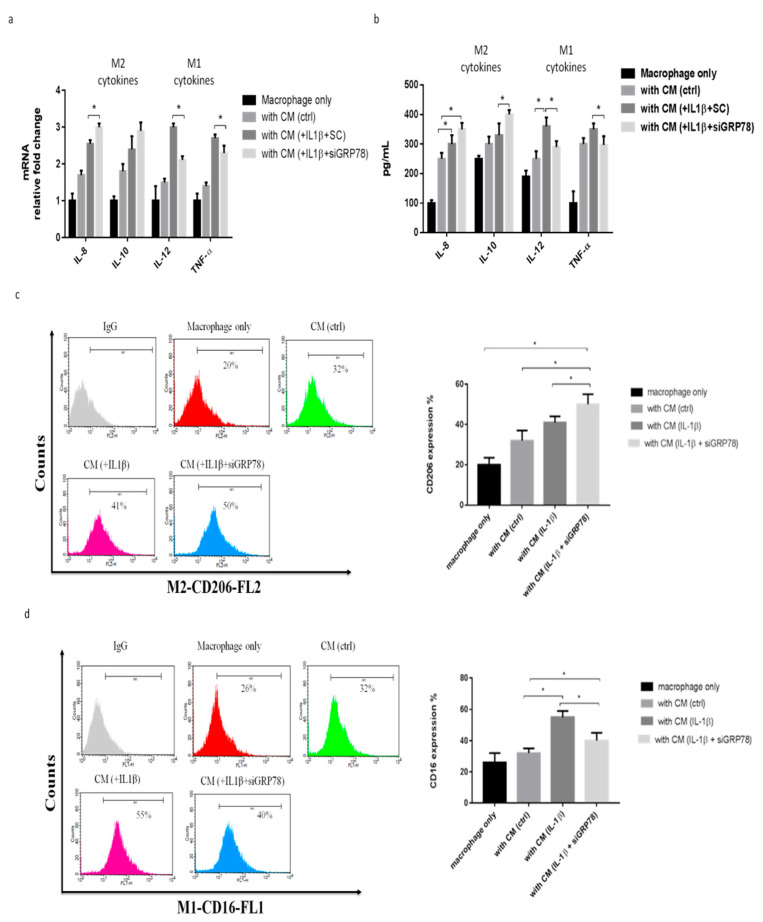
Knockdown GRP78 in synoviocytes regulated macrophage polarization to promote the M2 phenotype, using knockdown GRP78 in IL-1β (10 ng/mL) treated synoviocytes. Then, the CM was collected from synoviocytes to treat THP-1 cells. (**a**) Quantitative PCR analysis of IL-8, IL-10, IL-12, and TNF-α. (**b**) ELISA analysis of IL-8, IL-10, IL-12, and TNF-α. (**c**,**d**) Flow cytometry analysis of the expression of CD206, a marker of M2, and the expression of CD16, a marker of M1. Data are expressed as mean ± SD, * *p* < 0.05. IgG as a negative control. The data presented are representative of three independent experiments.

To explore whether GRP78 is involved in and regulates the IL-1β mediated inflammatory response in synoviocytes in OA, using siGRP78 in synoviocytes, we first showed the knockdown efficiency of GRP78 in synoviocytes (Figure 3a). The inflammatory genes IL-6, IL-8, substance P (SP), PGE-S, TNF-α, iNOS, and COX2 were significantly decreased in the group treated with a combination of IL-1β and siGRP78, compared with the group treated with IL-1β only (Figure 3b). The cytokine levels of IL-6 and PGE2 in synoviocytes were significantly decreased in the group treated with the combination of IL-1β and siGRP78 compared with the group treated with IL-1β only (Figure 3c,d). Moreover, the phosphorylation of p65 was decreased in those treated with the combination of IL-1β and siGRP78, compared with those treated with IL-1β only (Figure 3e). These findings indicated that the knockdown of GRP78 decreased the inflammatory factors and associated inflammatory signaling. We then collected the supernatant from synoviocytes as CM, which we used to treat the macrophages. We then examined the macrophage phenotypes. The M2 cytokines (IL-8 and IL-10) were significantly increased in the cells treated with CM plus IL-1β with siGRP78 compared with the CM plus IL-1β group (Figure 4a). By contrast, the M1 cytokines (IL-12 and TNF-α) were significantly decreased in the cells treated with CM plus IL-1β with siGRP78 compared with the CM plus IL-1β group (Figure 4a). Similarly, the M2 cytokines were also significantly increased in the cells treated with CM plus IL-1β with siGRP78 compared with the CM plus IL-1β group (Figure 4b). The M1 cytokines were also significantly decreased in the cells treated with CM plus IL-1β with siGRP78 (Figure 4b). In addition, the expression of CD206 (M2 surface marker) was increased in the cells treated with CM plus IL-1β with siGRP78, compared with the CM plus IL-1β group (Figure 4c), and the expression of CD16 (M1 surface marker) was decreased in the cells treated with CM plus IL-1β, compared with the CM group (Figure 4d). These data suggest that the knockdown of GRP78 can decrease the inflammatory response and regulate macrophage polarization to promote the M2 phenotype.

### 2.4. HMW-HA Treatment Improves Synovium Inflammation and Macrophage Polarization through GRP78 Expression

**Figure 5:**
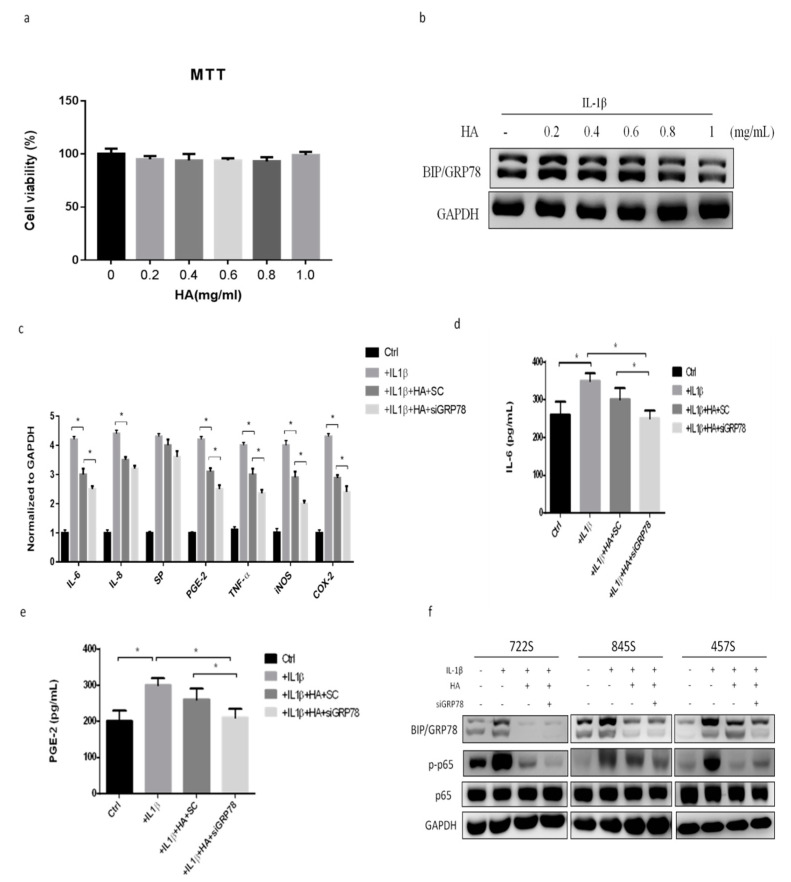
Effects of HMW-HA treatment on inflammatory factors and GRP78 in primary synoviocytes: (**a**) The primary synoviocytes were dose-dependently treated with HMW-HA for 24 h, and then, cell viability was assessed using the MTT assay. (**b**) The primary synoviocytes treated with IL−1β plus HMW-HA in a dose-dependent manner for 24 h and Western blot analysis of GRP78. (**c**) The primary synoviocytes were treated with IL−1β plus HMW-HA with siGRP78. Quantitative PCR analysis of IL−6, IL−8, SP, PGE−S, TNF−α, iNOS, and COX2. (**d**,**e**) ELISA analysis of IL−6 and PGE2. (**f**) Western blot analysis of GRP78, p65 phosphorylation, and p65. Data are expressed as a mean ± SD, * *p* < 0.05. The data presented are representative of three independent experiments.

**Figure 6:**
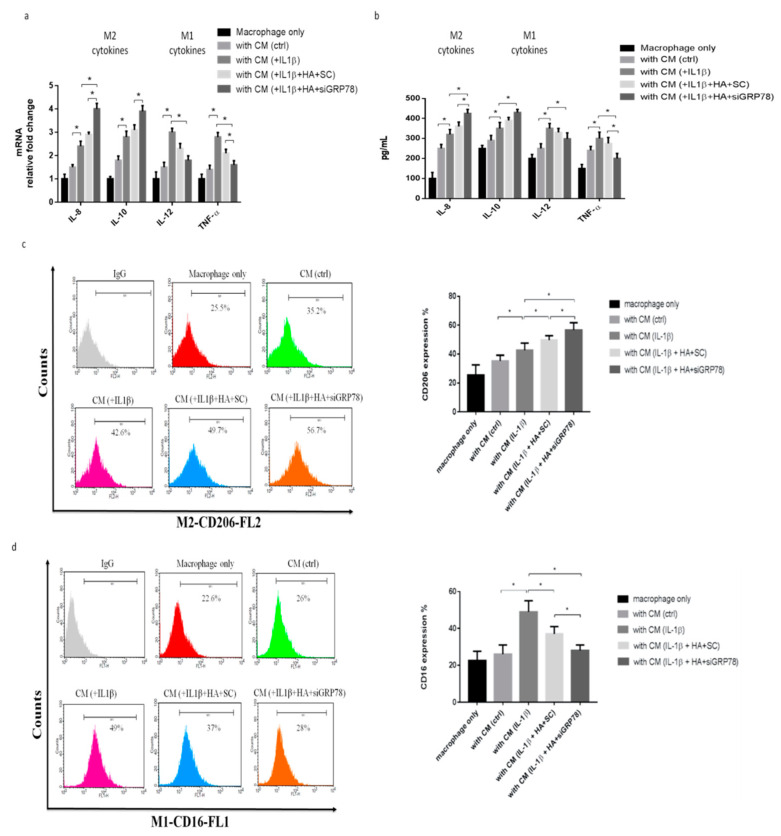
HMW-HA treatment in synoviocytes affected macrophage polarization toward the M2 phenotype. HMW-HA treated in knockdown GRP78 synoviocytes, and the supernatant was then collected from synoviocytes as CM and then used to treat THP-1. (**a**) Quantitative PCR analysis of IL-8, IL-10, IL-12, and TNF-α. (**b**) ELISA analysis of IL-8, IL-10, IL-12, and TNF-α. (**c**,**d**) Flow cytometry analysis of the expression of CD206, a marker of M2, and the expression of CD16, a marker of M1. Data are expressed as a mean ± SD, * *p* < 0.05. IgG as a negative control. The data presented are representative of three independent experiments.

**Figure 7:**
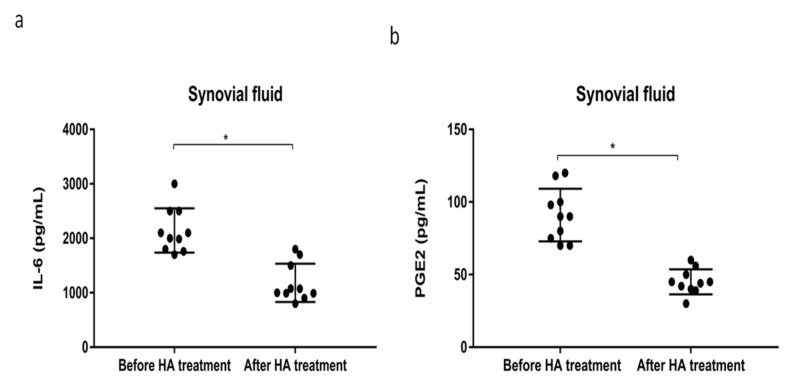
Effects of HMW-HA treatment on the inflammatory cytokines in the synovial fluid from OA patients. The inflammatory cytokines from the synovial fluid before and after HMW-HA treatment from 10 patients with OA: (**a**,**b**) ELISA analysis of IL-6 and PGE2. Data are expressed as a mean ± SD, * *p* < 0.05. The data presented are representative of three independent experiments.

HMW-HA (*M*_W_ 1000–2000 kDa) is used to treat OA, most commonly for OA of the knee. Next, we tried to explore the effect of HMW-HA treatment for synovial inflammation processes and macrophage polarization through GRP78 signaling in synoviocytes. First, we examined the viability of synoviocytes and GRP78 expression after treatment with different dosages of HMW-HA. We found that different HMW-HA dosages did not affect cell viability (Figure 5a), but GRP78 expression was decreased in a dose-dependent manner based on HMW-HA (Figure 5b). Second, we examined the inflammatory factors in synoviocytes after the HMW-HA treatment (1 mg/mL). The inflammatory genes IL-6, IL-8, SP, PGE-S, TNF-α, iNOS, and COX2 were slightly decreased after the HMW-HA treatment and were significantly more decreased with IL-1β plus HMW-HA in the siGRP78 group, compared with the group treated with IL-1β plus HMW-HA (Figure 5c). The cytokine levels of IL-6 and PGE2 in synoviocytes were slightly decreased after the HMW-HA treatment and were significantly more decreased in the IL-1β plus HMW-HA with siGRP78 group, compared with the IL-1β plus HMW-HA group (Figure 5d,e). Moreover, there was a significant decrease in the phosphorylation of p65 in the IL-1β plus HMW-HA group and the IL-1β plus HMW-HA with siGRP78 group as compared to the IL-1β group (Figure 5f). This result indicated that HMW-HA might suppress the inflammatory responses partially through GRP78. To investigate whether HMW-HA treatment can improve inflammatory responses and regulate macrophage polarization, we collected the supernatant from synoviocytes as CM, which we then used to treat macrophages and examine macrophage phenotypes. The M2 cytokines (IL-8 and IL-10) were slightly increased after the HMW-HA treatment and were significantly increased in the group treated with CM plus IL-1β with HMW-HA and siGRP78 compared with the CM plus IL-1β with HMW-HA group (Figure 6a). In contrast, the M1 cytokines (IL-12 and TNF-α) were slightly decreased after the HMW-HA treatment and were significantly decreased in the CM plus IL-1β with HMW-HA and siGRP78 group compared with the CM plus IL-1β with HMW-HA group (Figure 6a). Similarly, the M2 cytokines were slightly increased after the HMW-HA treatment and were also significantly increased in the CM plus IL-1β with HMW-HA and siGRP78 group compared with the CM plus IL-1β with HMW-HA group (Figure 6b). The M1 cytokines were slightly decreased after the HMW-HA treatment and were also significantly decreased in the CM plus IL-1β with HMW-HA and siGRP78 group compared with the CM plus IL-1β with HMW-HA group (Figure 6b). In addition, the expression of CD206 (an M2 surface marker) was increased in the CM plus IL-1β with HMW-HA group and was further increased in the CM plus IL-1β with HMW-HA and siGRP78 group, compared with the CM plus IL-1β with HMW-HA group (Figure 6c), and the expression of CD16 (an M1 surface marker) was decreased in the CM plus IL-1β with HMW-HA group and was further decreased in the CM plus IL-1β with HMW-HA and siGRP78 group, compared with the CM plus IL-1β with HMW-HA group (Figure 6d). Finally, we analyzed the inflammatory cytokines in the synovial fluid before and after the HMW-HA treatment from OA patients. The levels of the cytokines IL-6 and PGE2 were significantly decreased after the HMW-HA treatment (Figure 7a,b). The above results showed that the HMW-HA treatment decreased the GRP78 expression in synoviocytes and then affected the inflammatory cytokines and macrophage polarization toward the M2 phenotype.

## 3. Discussion

Previous studies have shown that IL-1β induces an unfolded protein response and that it is associated with cartilage and synovial cells during the induction of OA, such as C/EBP homologous protein (CHOP) and X-box binding protein 1 (XBP1) [27,28]. XBP1 and CHOP increase the release of matrix metallopeptidase (MMP-3) and the related inflammatory cytokines, resulting in the inflammation of the synovial cells and the degradation of chondrocytes [12]. However, under some experimental conditions [28], XBP1s also have a beneficial effect on the survival of chondrocytes. An in vivo surgically induced instability model of knee OA in mice revealed that the knockout of CHOP partially protected against chondrocyte apoptosis and cartilage degradation [29]. In this study, we first found that the GRP78 in synovial cells showed an increasing trend in an IL-1β-induced mimic OA microenvironment. The increased expression of GRP78 was also accompanied by an increase in the secretion of inflammation-related cytokines and activation of the inflammatory pathway. The elimination of GRP78 can reduce the inflammatory condition of synovial cells. The NF-κB pathway was most prominent in human disease and OA models, suggesting a key regulatory role for stress and inflammatory signaling via the NF-κB pathway in OA [30,31]. Abnormal NF-κB pathways cause a loss of chondrocyte growth arrest, and they also produce pro-degrading hormones, including aggrecanase and MMP, which induce cartilage degradation, as well as proinflammatory cytokines [31]. The results of this study show that knockdown GRP78 in the synovial cells reduced the phosphorylation of p65, indicating that GRP78 affected the NF-κB inflammatory pathway and then the proinflammatory cytokines.

Recent studies have shown that macrophages play an important role in the mechanism of OA and RA. In OA, synovial inflammation and cartilage destructiveness were associated with the involvement of macrophages, and these effects were mediated through the cytokines IL-1β and TNF-α [32]. A previous study used a mouse model induced by clodronate liposome treatment to deplete the macrophages of the synovial lining, which resulted in a significant reduction in osteophyte formation as well as in TGF-β, BMP-2, and BMP-4, which are thought to be implicated in osteophyte formation [33]. Numerous in vivo and in vitro studies have since suggested that macrophage polarization from M1 to M2 and vice versa could be a potential treatment strategy for a number of diseases [34]. The studies in RA, OA, gout synovitis, and spondyloarthritis indicate that the balance between M1 and M2 macrophages significantly affects synovial inflammation [35,36]. Synovial macrophages polarized into M1 through the expression of R-spondin-2 (Rspo2) cause OA [37]. In a high-fat-diet-induced obesity mice model of OA, it was also found that the M1 phenotype was increased [38]. In clinical studies, the ratio of M1/M2 in the synovial fluid of patients with OA was higher than that in healthy individuals [39]. In recent years, the relationship between ER stress and polarization of macrophages has been found to be correlated [40]. One study showed that ER stress inositol-requiring enzyme 1α (IRE1α) inhibited the polarization of a macrophage into M2 and then impaired energy expenditure in obesity [41], and the knockdown of CHOP in adipocytes was found to increase the polarization of a macrophage into M2 [42]. However, studies on the relationship between ER stress and the polarization of synovial macrophages are still lacking. In this study, we first found that increased GRP78 expression in IL-1β-induced synovial cell inflammation caused the production of related inflammatory factors, which, in turn, affected the polarization of macrophages. Furthermore, although we knocked down GRP78, the related inflammatory factors were decreased, and the macrophages were polarized into M2.

Injecting HA into the joint is still one way to treat OA. The traditional mechanism is to provide viscoelasticity to the synovial fluid, reducing friction and stress. HA can reduce the destruction of chondrocytes by absorbing internal pressure and vibration [43], and HMW-HA can reduce friction and achieve a therapeutic effect as a result of its greater viscosity [43]. Studies have shown that HA treatment can reduce the IL-1β-induced inflammatory response and result in anti-inflammatory effects [44]. The HA treatment of OA is typically conducted by inhibiting the production of inflammatory cytokines, including IL-8, IL-6, and PGE2 [8]. Other studies have shown that HA of different molecular weights affects the polarization of macrophages. HMW-HA increases the M2 phenotype, whereas HA of a low molecular weight increases the M1 phenotype [45]. One previous study indicated that, in ER stress, inflamed cells bind to the HA-rich extracellular matrix and increase chronic inflammation [46]. However, only a few studies have focused on the interaction between HMW-HA and ER stress regulators. In our study, we found that HMW-HA can inhibit the expression of GRP78, which causes the inflammatory factors to be altered in synovial cells, hence affecting the polarization of macrophages. Moreover, after treatment with both HMW-HA and siGRP78, we found a significantly synergistic effect of reducing the activation of related inflammatory factors and inflammatory pathways and increasing the M2/M1 ratio. Our clinical synovial fluid samples also confirmed the in vitro data, indicating that HMW-HA treatment can reduce inflammatory cytokines, especially IL-6 and PGE-2 levels. Transmembrane protein 2 (TMEM2) can degrade HA and increase the ER stress expression [47], whereas ER stress also induces HA deposition that is conducive to leukocyte binding [46]. CD44 is the major cell surface hyaluronan receptor that is highly expressed by alveolar macrophages. M1 to M2 polarization occurs due to the high affinity of HA to the CD44 receptors of macrophages [48]. Consistent with our results, the HA treatment of synoviocytes affects the macrophages’ phenotype forward M2, and it does so through the regulation of the ER stress marker, GRP78. TGF-β stimulated HA production and reduced NF-kB activation and apoptosis in human fibroblast [49]. The study indicated cross talk among GRP78 and NF-κB in prostate cancer cells [50], and the abrogation of GRP78 blunts the activation of NF-κB through the ATF6 branch of the UPR [51]. Blocking the activation of NF-κB signaling can promote the polarization of M1 macrophages to M2 macrophages [21]. Consistent with our results, the HA treatment reduced GRP78 and NF-κB, and increased M2 macrophages.

Our study had limitations. First, IL-1β was used to induce OA, but IL-1β can also indirectly cause p65 phosphorylation; therefore, this part should have used a p65 phosphorylation inhibitor to verify the role of IL-1β in synovial cells. Second, we used the over-expression of GRP78 in primary synovial cells to evaluate the effects on an IL-1β-induced inflammatory response and macrophage polarization, as well as the relatively significant effects of GRP78 knockdown in our study. Third, HA alone can also inhibit the expression of inflammatory genes and p-p65, while the combined use of HMW-HA and siGRP78 can enhance the inhibitory effect, but some still need more experiments to confirm this. Fourth, in vivo animal experiments would be necessary in the future to support the in vitro data in our study.

In conclusion, the present study elucidates the role of GRP78 in OA-related synovial inflammation and macrophage polarization. In addition, our study provides a new vision of HMW-HA for OA treatment. Our results indicate the possible mechanism by which HMW-HA can reduce synovial inflammation through the inhibition of the GRP78/NF-κB inflammatory pathway and proinflammatory cytokines, thereby affecting the polarization of synovial macrophages, increasing the polarization of M2, and achieving anti-inflammatory effects.

## 4. Materials and Methods

### 4.1. Cell Culture and Reagents

The THP-1 cell line was obtained from the Bioresource Collection and Research Center (Taipei, Taiwan) and maintained in RPMI-1640 medium (Gibco, Grand Island, NY, USA) supplemented with 10% fetal bovine serum (FBS; Gibco BRL, Grand Island, NY, USA), 100 U/mL penicillin (Thermo, Wilmington, DE, USA), and 100 U/mL streptomycin (Thermo, Wilmington, DE, USA) under 5% CO_2_ at 37 °C. THP-1 cells were differentiated by 200 nM PMA for 24 h (phorbol 12-myristate 13-acetate; Sigma-Aldrich, St. Louis, MO, USA) [52]. The reagents IL-1β (PeproTech, Cranbury, NJ, USA) and HMW-HA (Acros Organics, Cranbury, NJ, USA) were obtained for in vitro studies.

### 4.2. Source and Culture of Human Primary Synoviocytes

Human synovial tissue samples were obtained from knee joint patients with OA undergoing a total knee replacement (*n* = 39, mean age, 70 years; range 58–82 year). All experimental protocols were approved by Taipei Medical University (IRB-CRC-01-10-03), and all patients gave written informed consent. Synovial fluids were obtained by needle aspiration from patients before and after an HMW-HA (Hya-Joint Synovial Fluid Supplement (SciVision Biotech Inc., Kaohsiung, Taiwan) with 650–1200 kDa.) injection and then centrifuged at 14,000× *g* for 20 min. Supernatants were stored at −80 °C until use. For human primary synoviocytes, synovial tissues were minced, stirred with 3 mg/mL of blend collagenase type H (Sigma) in serum-free DMEM (Gibco) for 6 h before filtering through nylon mesh, and washing extensively. The synoviocytes were maintained in DMEM, supplemented with 10% FBS (Sigma), 100 I.U./mL penicillin (Gibco), and 100 μg/mL streptomycin (Gibco). Cells between passages 3 and 5 were used. In this study, 722S, 845S, and 457S were mainly used, all from different patients [53].

### 4.3. Preparation of Conditioned Medium (CM) from Synoviocytes

The synoviocytes were seeded at a density of 1 × 10^4^ cells/cm^2^ for 72 h. When cultures reached 80% to 90% confluence, cells were treated with IL-1β and HA. The medium was then replaced with fresh serum-free medium for 24 h, and then, cells were pelleted, first at low-speed centrifugation (250× *g* at 4 °C for 10 min), and then using high-speed centrifugation (1000× *g* at 4 °C for 10 min) to remove cell debris [54]. This was followed by filtration with a 0.22-mm filter (Millipore, Billerica, MA, USA). The CM was preserved at −80 °C for further study [55].

### 4.4. Quantitative Polymerase Chain Reaction Analysis

Total RNA was isolated using Trizol (Invitrogen, Grand Island, NY, USA). Complementary DNA synthesis was performed using a SuperScript^®^ III Reverse Transcriptase kit (Invitrogen, Grand Island, NY, USA). We then performed a real-time polymerase chain reaction (PCR) for the gene of interest and SYBR green dye (Thermo, Wilmington, DE, USA) using the LightCycler^®^ 480 System (Roche). The reaction mixture containing reverse-transcribed cDNAs was preheated for 2 min at 95 °C to activate the Taq polymerase. Forty cycles of PCR, each consisting of a 10-s denaturation step at 95 °C and a 30-s annealing step at 60 °C (two-step real-time (RT)-PCR), were then performed. Throughout the RT-PCR analysis, product identities were confirmed using melting curve analysis. The ratio of the amounts of target mRNA to the amount of the internal standard (GAPDH) mRNA was determined as an arbitrary unit [52].

### 4.5. Western Blotting

Cell lysates were harvested in an RIPA buffer (1% sodium dodecyl sulfate (SDS) and 10 mM Tris buffer pH 7.4) containing protease inhibitors and a phosphatase inhibitor (Thermo, Wilmington, DE, USA). Protein concentrations of the supernatants were determined using the Pierce BCA Protein Assay Kit (Thermo, Rockford, IL, USA). Thirty micrograms of protein was separated on a 5% to 15% gradient SDS–polyacrylamide gel electrophoresis gel and transferred to polyvinylidene difluoride membranes (Millipore, Bedford, MA, USA) by wet blotting using an electroblotter (Hoefer system). Membranes were blocked for 1 h at 25 °C with 5% skim milk in Tris buffered saline with Tween 20 (TBST). The membranes were incubated with appropriate dilutions of the following primary antibodies: BIP/GRP78 antibody (ab32618 (1:1000 dilution); Abcam, Cambridge, UK), phospho-p65 antibody (#8241 (1:1000 dilution); Cell Signaling, Danvers, MA, USA), p65 antibody (#3033 (1:1500 dilution); Cell Signaling, Danvers, MA, USA), and GAPDH antibody (#5174 (1:1000 dilution); Cell Signaling, Danvers, MA, USA) overnight at 4 °C. After being washed in TBST three times, the membranes were incubated for 60 min with horseradish peroxidase-conjugated goat anti-rabbit or anti-mouse secondary antibodies at 25 °C. Specific bands were detected using chemiluminescence, and visualization was performed by exposing the membranes to a UVP imaging system (Analytik Jena, Upland, CA, USA) [56].

### 4.6. siRNA Transfection

GRP78 expression was suppressed in synoviocytes using small-interfering RNA (siRNA). siGRP78 and control siRNA were purchased from Dharmacon’s Smartpool siRNA (Lafayette, CO, USA), and PolyJet In Vitro DNA Transfection Reagent (SignaGen, Frederick, MD, USA) was used according to the manufacturer’s protocol [57].

### 4.7. Enzyme-Linked Immunosorbent Assay (ELISA)

The THP-1 cells (5 × 10^4^ cells per well of a 96-well plate) were incubated with CM. After a 48-h incubation, the supernatants were collected, followed by the determination of human IL-8, IL-10, IL-12, and TNF-α using the Ready-Set-Go ELISA kit (eBioscience, San Diego, CA, USA) according to the manufacturer’s instructions. The synoviocytes (5 × 10^4^ cells per well of a 96-well plate) were treated with IL-1β and HA. After treatment for 48 h, the supernatants were collected, followed by the determination of human IL-6 and PGE2 assay kit (R&D Systems, Minneapolis, MN, USA) according to the manufacturer’s instructions.

### 4.8. Fluorescence-Activated Cell Sorter Analysis

The THP-1 cells (1 × 10^6^ cells per well of a six-well plate) were incubated with CM. After a 48-h incubation, cells were collected using a scraper and blocked with an incubation buffer (dissolved in 0.5 g of bovine serum albumin in 100 mL of phosphate-buffered saline) for 45 min and then incubated with an FITC-conjugated CD16 antibody (11-0168 (1:100 dilution) eBioscience, San Diego, CA, USA) and a PE-conjugated CD206 antibody (12-2069 (1:100 dilution); eBioscience, San Diego, CA, USA) for 60 min at 25 °C. Following the final washing step, labeled cells were analyzed using flow cytometry on a FACScan flow cytometer using CellQuest software (Becton-Dickinson, Franklin Lakes, NJ, USA). The total number of cells to be harvested was set at 2 × 10^5^, and the collection speed was controlled at 200 to 300 cells/s.

### 4.9. Statistical Analysis

Data are expressed as the mean ± SD, unless otherwise noted. The differences between groups were analyzed using a two-tailed Student’s *t* test when only two groups were present, and the null hypothesis was rejected at the 0.05 level.

## Data Availability

The data presented in this study were available on request from the corresponding author.
